# Age-Related Utilization of Thrombus Aspiration in Patients With ST-Segment Elevation Myocardial Infarction: Findings From the Improving Care for Cardiovascular Disease in China Project

**DOI:** 10.3389/fcvm.2022.791007

**Published:** 2022-02-21

**Authors:** Yang-Yang Qu, Xiao-Guo Zhang, Cheng-Wei Ju, Ya-Min Su, Rui Zhang, Wen-Jie Zuo, Zhen-Jun Ji, Li-Juan Chen, Gen-Shan Ma

**Affiliations:** Department of Cardiology, School of Medicine, Zhongda Hospital, Southeast University, Nanjing, China

**Keywords:** thrombus aspiration, primary percutaneous coronary intervention, ST-segment elevation myocardial infarction, age, adverse cardiac events, stroke

## Abstract

**Background:**

There are some controversies on the utilization and benefits of thrombus aspiration in patients with ST-segment elevation myocardial infarction (STEMI). However, a few studies investigated this issue and the age-associated effects among the large population in China. Hence, we aimed to figure out the age-associated utilization and in-hospital outcomes of thrombus aspiration to improve therapeutic decisions in clinical routine.

**Methods:**

We retrospectively recruited 13,655 eligible STEMI patients from the database of the Improving Care for Cardiovascular Disease in China-Acute Coronary Syndrome project. These subjects were allocated into primary percutaneous coronary intervention (PPCI)-only group and thrombus aspiration group after being subdivided into three age groups (G_21−50_, G_51−75_, and G_76−95_). After 1:1 propensity score matching for PPCI-only and thrombus aspiration groups, a total of 8,815 matched patients were enrolled for the subsequent analysis. The primary outcome was in-hospital cardiovascular death, and the key safety outcome was in-hospital stroke.

**Results:**

We observed that the ratio of STEMI patients undergoing thrombus aspiration to PPCI-only reduced with aging. For patients ≤ 75 years, the culprit lesion suffered from thrombus aspiration was mainly located in the left anterior descending branch, and left-ventricular ejection fraction (LVEF) was lower (G_21−50_: 54.9 ± 8.9 vs. 56.0 ± 8.7%, *P* = 0.01; G_51−75_: 53.9 ± 9.6 vs. 54.8 ± 9.0%, *P* = 0.001) and the rate of regional wall motion abnormality was higher (G_21−50_: 75.7 vs. 66.5%, *P* < 0.001; G_51−75_: 75.4 vs. 69.1%, *P* < 0.001) in the thrombus aspiration group. By contrast, for patients > 75 years, the right coronary artery was the predominant culprit lesion undergoing thrombus aspiration, LVEF (63.1 ± 10.5 vs. 53.1 ± 9.5%, *P* = 0.985) and the regional wall motion abnormality (79.2 vs. 74.2%, *P* = 0.089) were comparable between the two treatment groups. Thrombus aspiration neither reduced the in-hospital risk of cardiovascular death, all-cause death, recurrent myocardial infarction, acute stent thrombosis, heart failure, cardiogenic shock, and sudden cardiac arrest nor increased stroke risk compared with the PPCI-only group. However, after adjustment for age, thrombus aspiration presented the tendency to reduce the incidence of sudden cardiac arrest (4.9 vs. 2.5%, *P* = 0.06) and in-hospital cardiovascular death at 3 days (hazard ratio 0.46; 95% CI, 0.20–1.06; log-rank *P* = 0.08) in G_76−95_ group and tended to increase the incidence of heart failure in G_51−75_ (5.7 vs. 6.9%, *P* = 0.07).

**Conclusion:**

The thrombus aspiration neither significantly reduced the in-hospital incidence of major adverse cardiac events nor increased stroke risk. However, it might play a protective role in reducing in-hospital sudden cardiac arrest and increasing survival from cardiovascular death at 3 days for the elderly.

## Background

ST-segment elevation myocardial infarction (STEMI) is a severe cardiovascular disease, the major cause of which is a complete coronary artery occlusion due to the formation of the thrombus (1). Primary percutaneous coronary intervention (PPCI) within the first 12 h of the symptom onset has been proven as the most efficient therapeutic means to reperfuse the infarcted myocardium in the clinical routine (1, 2). However, the manipulation of atherectomy catheters, balloons, and stents during the PPCI procedure has the chance to cause distal embolization of thrombus. The subsequent obstructed microvasculature, no-reflow phenomenon, impaired tissue perfusion and increased infarct size may lead to cardiovascular death (3).

The routine use of manual thrombus aspiration during the percutaneous coronary intervention (PCI) could improve the primary outcome of microvascular perfusion at 30 days and decrease 1-year cardiac death and non-fatal reinfarction, which was reported by Thrombus Aspiration during Percutaneous Coronary Intervention in Acute Myocardial Infarction Study (TAPAS) (4, 5). However, there were some debates on this issue. Thrombus Aspiration in ST-Elevation Myocardial Infarction in Scandinavia (TASTE trial) revealed that thrombus aspiration could not significantly reduce the mortality either at 30 days or 1 year (6, 7). As a larger registry enrolling 10,732 patients at 87 hospitals in 20 countries, Trial of Routine Aspiration Thrombectomy with PCI vs. PCI Alone in Patients with STEMI (TOTAL) disclosed that thrombus aspiration could not reduce the 180-day and 1-year risk of cardiovascular death, recurrent myocardial infarction, cardiogenic shock, or heart failure. However, the rates of stroke within 30 and 180 days were higher in the thrombus aspiration group (2, 8). Even though thrombus aspiration was not recommended as a routine procedure by TOTAL and TATSE trials, it is considered in case of a large residual thrombus burden (9, 10).

To figure out the nationwide utilization and clinical outcomes of thrombus aspiration to provide evidence for therapeutic strategy in clinical routine, we analyzed the real-world data from the Improving Care for Cardiovascular Disease in China–Acute Coronary Syndrome (CCC–ACS) project, which is the largest project and quality improvement registry program for ACS in China (11). Considering the utilization of thrombus aspiration differed for patients at different ages, age subgroups were established for the subsequent investigation.

## Methods

### CCC–ACS Project and Data Collection

CCC–ACS project was co-launched by the American Heart Association (AHA) and Chinese Society of Cardiology (CSC) and collected the information of ACS patients. Unlike TAPAS, TASTE, and TOTAL, which were prospective trials (12–14), the CCC–ACS project did nothing to intervene in the treatment in clinical practice. The principal purpose of this project was to describe the baseline characteristics, in-hospital treatment, and outcomes of ACS patients in China, and then make efforts to optimize the therapeutic strategies and improve therapeutic efficacy accordingly (15). A total of 150 tertiary hospitals from different geographic and economic regions were recruited. The detailed rationale and design of this project have been published before (15). Briefly, in each hospital, the information of the first 20–30 ACS patients was consecutively reported by a well-trained physician on a web-based data collection platform (Oracle Clinical Remote Data Capture; Oracle Corporation, Redwood City, CA, United States) on the official website (www.ccc-heart.com) month by month (11). The comprehensive information includes demography, medical history, risk factors, symptoms on arrival, in-hospital laboratory results, reperfusion, medication treatment, and events, as well as medications and counseling at discharge. This project included both unstable angina pectoris and acute myocardial infarction cases, acute myocardial infarction cases have reporting priority. Finally, 63,641 patients diagnosed as the ACS based on the symptoms such as chest pain or distress, alterations of myocardial injury biomarkers, and anomalous results of an electrocardiogram (16, 17) were enrolled from November 2014 to July 2017.

To ensure the accuracy of data collection, the data collection platform was equipped with automatic checks for invalid values. Moreover, the clinical research associates were designated to perform a regular on-site quality inspection. A 5% of reported cases would be randomly selected for the comparisons between original medical records and reported data. This study was approved by the Ethics Committee of Beijing Anzhen Hospital, Capital Medical University, and it has been registered at www.ClinicalTrials.gov (Unique identifier: NCT02306616).

### Recruitment of Study Population

STEMI was defined according to the guideline issued by the CSC in 2010 (16). In this study, the inclusion criteria of patients were (i) undergoing PPCI within 12 h of the onset of the symptoms and (ii) placement of at least one drug-eluting stent (DES). The exclusion criteria were (i) placement of a bare-metal stent; (ii) subject to coronary artery bypass graft surgery before; (iii) undergoing fibrinolytic therapy at admission; and (iv) lacking information on age, thrombus aspiration treatment (yes / no), or Killip classification. Considering the potential age-associated utilization and dissimilarity of clinical outcomes of thrombus aspiration, the recruited subjects were divided into three age subgroups: G_21−50_ (range 21–50 years), G_51−75_ (range 51–75 years), and G_76−95_ (range 76–95 years) for the subsequent analysis.

### Study Outcomes

The occurrence of in-hospital major adverse cardiac cerebrovascular events was evaluated in this study. The primary efficacy outcome was cardiovascular death. The secondary outcomes were all-cause death, recurrent myocardial infarction, acute stent thrombosis, heart failure, cardiogenic shock and sudden cardiac arrest. The key safety outcome was stroke during hospitalization. LV ejection fraction (LVEF) and regional wall motion were examined with echocardiography before discharge.

### Statistical Analysis

Categorical variables were presented as numbers and percentages. Continuous variables were expressed as mean ± SD or median (interquartile ranges), as appropriate. A chi-squared test was used to evaluate the differences for categorical variables. Unpaired *t*-test or Mann–Whitney *U*-test was used to test the differences of continuous variables between PPCI-only and thrombus aspiration groups, as applicable. The connection between patients' age and thrombus aspiration treatment was assessed with a logistic regression model and expressed as OR with 95% CI. All recruited patients were matched between PPCI-only and thrombus aspiration groups with 1:1 propensity score matching in each age subgroup to diminish the influence of confounders. The prespecified covariates used to calculate propensity scores were age, gender, heart rate, systolic blood pressure (SBP), Killip classification, smoking, medical history (prior myocardial infarction, prior PCI, heart failure, hypertension, hyperlipemia, diabetes mellitus, renal failure, cerebral infarction, and cerebral hemorrhage), culprit lesions (left main coronary artery [LM], left anterior descending branch [LAD], left circumflex branch [LCX], right coronary artery [RCA]), in-hospital medications (dual antiplatelet therapy [DAPT, aspirin, and clopidogrel/ticagrelor], angiotensin-converting enzyme inhibitor (ACEI) or angiotensin receptor blocker [ARB], statin, β receptor blocker, glycoprotein IIb/IIIa [GP IIb/IIIa] inhibitor, and low molecular heparin), as well as gross domestic product. The match tolerance was set as 0.02. For variables with missing data, we imputed the missing values with the sequential regression multiple imputation method implemented by the IVEware software version 0.2 (Survey Research Center, University of Michigan, Ann Arbor, MI, United States). The Cox proportional hazards regression model was used to evaluate time-to-event data of all adverse cardiovascular events after the adjustment of age, and then generate hazard ratio (HR) and 95% CIs. Kaplan–Meier analysis was performed for assessing survival from in-hospital cardiovascular death at 3, 7, and 10 days, and the statistical significance was tested with the log-rank test. In all cases, two-tailed tests were applied and significance was determined as *P* < 0.05.

## Results

### Recruitment of Study Population

**Figure 1:**
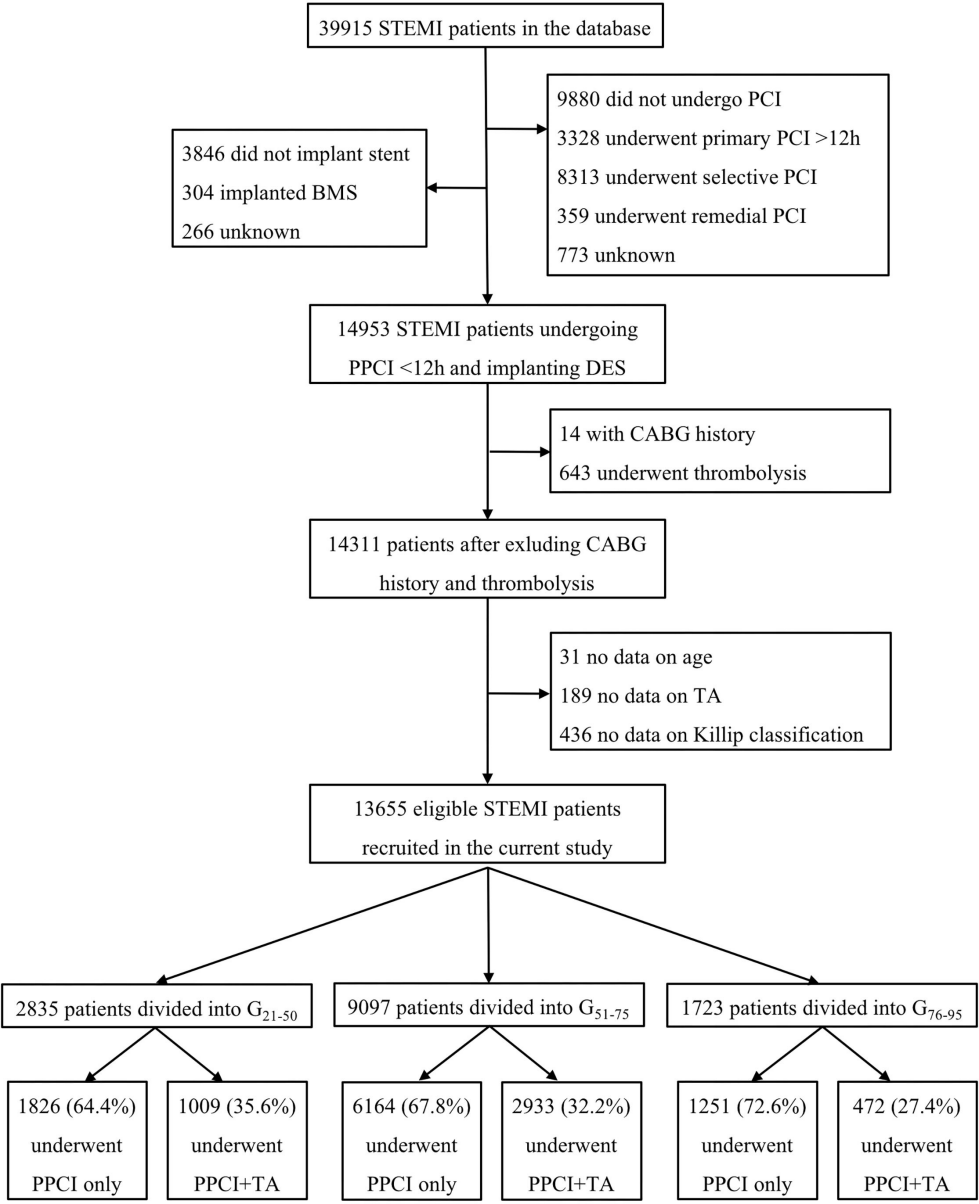
Study flowchart. STEMI, ST-segment elevation myocardial infarction; PCI, percutaneous coronary intervention; PPCI, primary percutaneous coronary intervention; BMS, bare metal stent; DES, drug-eluting stent; CABG, coronary artery bypass grafting; TA, thrombus aspiration.

A total of 63,641 ACS patients were reported in the database from November 2014 to July 2017, of whom 39,915 (62.7%) were diagnosed as STEMI; 14,953 of 39,915 (37.5%) STEMI patients underwent PPCI treatment within 12 h of the onset of symptoms and implanted at least one DES in the culprit lesion. After excluding patients with coronary artery bypass grafting history, undergoing fibrinolytic therapy at admission, or lacking information on age, thrombus aspiration and Killip classification, 13,655 eligible patients were enrolled for this study (Figure 1).

### Age-Associated Utilization of Thrombus Aspiration

**Figure 2:**
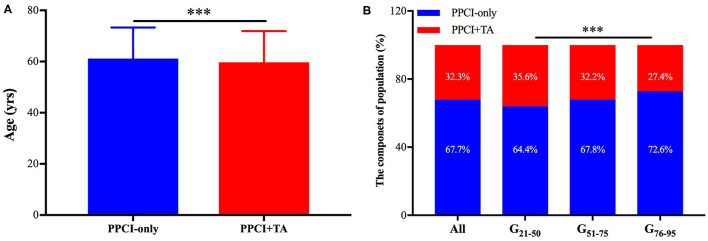
The age-associated utilization of thrombus aspiration. **(A)** The patients in the PPCI-only group were significantly older than those in PPCI combining thrombus aspiration group. ^***^P < 0.001. **(B)** The percentages of STEMI patients undergoing PPCI-only and thrombus aspiration were presented. PPCI, primary percutaneous coronary intervention; TA, thrombus aspiration; CI, confidence interval.

Among the whole enrolled population, the patients in the PPCI-only group were significantly older than those in PPCI combining thrombus aspiration group (61.2 ± 12.1 years vs. 59.7 ± 12.2 years, *P* < 0.001) (Figure 2A). Logistic regression analysis also revealed that thrombus aspiration was less likely to be conducted with the increase of age (OR = 0.990; 95% CI, 0.987 to 0.993; *P* < 0.001) (Supplementary Figure 1). We divided the patients into three age subgroups for the subsequent analysis. The patients between 51 and 75 years accounted for a substantial part of the study population (*n* = 9,097, 66.6%). The proportions of patients ≤ 50 years and >75 years reached 20.8% (*n* = 2,835) and 12.6% (*n* = 1,723), respectively. The percentages of patients undergoing thrombus aspiration in G_21−50_ and G_51−75_ groups were discernibly higher than that in the G_76−95_ group (35.6% in G_21−50_, 32.2% in G_51−75_, and 27.4% in G_76−95_, *P* < 0.001), in which the percentage of STEMI patients undergoing PPCI only was almost 1.6 times higher than that of patients undergoing thrombus aspiration (Figure 2B).

### Clinical Characteristics of the Study Population

Age, the proportion of males, body mass index, and heart rate were comparable between PPCI-only and thrombus aspiration groups except for patients in the G_51−75_ subgroup (age: 62.4 ± 6.7 years *vs*. 62.0 ± 6.8 years, *P* = 0.020). The systolic and diastolic blood pressure were higher in the PPCI-only group. The percentage of Killip Class I decreased and the percentage of Killip classes II–IV increased with aging. The most frequently encountered risk factors for the development of STEMI were smoking and hypertension in the whole population. In addition, G_76−95_ patients showed a lower prevalence of smoking history and hyperlipemia as well as a higher incidence of prior myocardial infarction, PCI history, hypertension, diabetes mellitus and stroke compared with the youngest patients. In the G_21−50_ subgroup, the medical history was well-balanced between the two groups except for the proportion of smokers (71.8% in the PPCI-only group *vs*. 68.0% in the thrombus aspiration group, *P* = 0.036) (Table 1).

**Table 1:** The baseline characteristics of the patients (*n* = 13,655).

	**G**_**21−50**_			**G**_**51−75**_			**G**_**76−95**_		
	**PPCI-only** **(*n* = 1,826)**	**PPCI+TA** **(*n* = 1,009)**	***P*** **-value**	**PPCI-only** **(*n* = 6,164)**	**PPCI+TA** **(*n* = 2,933)**	***P*** **-value**	**PPCI-only** **(*n* = 1,251)**	**PPCI+TA** **(*n* = 472)**	***P*** **-value**
**BASELINE INFORMATION**									
Age (years)	44.0 ± 5.2	43.5 ± 5.8	0.067	62.4 ± 6.7	62.0 ± 6.8	**0.020**	80.4 ± 3.7	80.2 ± 3.7	0.348
Male, n (%)	1,748 (95.7%)	970 (96.1%)	0.602	4,959 (80.5%)	2,398 (81.8%)	0.138	780 (62.4%)	295 (62.5%)	0.954
BMI (kg/m^2^)	25.4 ± 3.3	25.2 ± 3.4	0.351	24.4 ± 3.0	24.5 ± 2.9	0.111	23.4 ± 3.0	23.5 ± 3.1	0.653
Heart Rate (bpm)	81.0 ± 15.2	81.2 ± 15.2	0.535	77.1 ± 15.9	76.8 ± 15.7	0.680	76.7 ± 17.2	76.2 ± 18.0	0.693
SBP (mmHg)	129.0 ± 22.7	127.0 ± 22.6	**0.019**	127.6 ± 23.6	123.9 ± 23.1	**<0.001**	126.8 ± 25.5	123.6 ± 24.1	**0.027**
DBP (mmHg)	82.0 ± 16.2	80.9 ± 16.0	**0.035**	78.0 ± 14.6	76.2 ± 14.6	**<0.001**	72.9 ± 14.7	72.3 ± 13.8	0.543
Killip class									
I, n (%)	1,485 (81.3%)	843 (83.5%)	0.155	4,752 (77.1%)	2,329 (79.4%)	**0.013**	866 (69.2%)	327 (69.3%)	0.982
II, n (%)	266 (14.6%)	129 (12.8%)	0.189	1,070 (17.4%)	437 (14.9%)	**0.003**	274 (21.9%)	105 (22.2%)	0.878
III, n (%)	24 (1.3%)	13 (1.3%)	0.958	125 (2.0%)	49 (1.7%)	0.245	39 (3.1%)	15 (3.2%)	0.949
IV, n (%)	51 (2.8%)	24 (2.4%)	0.510	217 (3.5%)	118 (4.0%)	0.234	72 (5.8%)	25 (5.3%)	0.713
**MEDICAL HISTORY**									
Smoking	1,241 (68.0%)	724 (71.8%)	**0.036**	3,188 (51.7%)	1,542 (52.6%)	0.446	340 (27.2%)	130 (27.5%)	0.880
Prior myocardial infarction	60 (3.3%)	25 (2.5%)	0.227	268 (4.3%)	136 (4.6%)	0.532	59 (4.7%)	19 (4.0%)	0.538
Prior PCI	59 (3.2%)	26 (2.6%)	0.328	281 (4.6%)	143 (4.9%)	0.503	74 (5.9%)	23 (4.9%)	0.402
Hypertension	668 (36.6%)	378 (37.5%)	0.642	3,026 (49.1%)	1,474 (50.3%)	0.299	718 (57.4%)	267 (56.6%)	0.757
Hyperlipemia	127 (7.0%)	87 (8.6%)	0.108	384 (6.2%)	212 (7.2%)	0.072	58 (4.6%)	27 (5.7%)	0.354
Diabetes Mellitus	236 (12.9%)	136 (13.5%)	0.676	1,288 (20.9%)	599 (20.4%)	0.603	240 (19.2%)	88 (18.6%)	0.799
Stroke	38 (2.1%)	20 (2.0%)	0.859	475 (7.7%)	258 (8.8%)	0.074	149 (11.9%)	61 (12.9%)	0.566

*Results are reported as mean ± SD or n (%). PPCI, primary percutaneous coronary intervention; TA, thrombus aspiration; BMI, body mass index; SBP, systolic blood pressure; DBP, diastolic blood pressure. The bold values indicate that the values smaller than 0.5 implying significant difference*.

The culprit lesion differed between PPCI-only and thrombus aspiration groups. Meanwhile, the culprit lesion undergoing thrombus aspiration was more likely to be LAD in G_21−50_ and G_51−75_ subgroups, and RCA in the G_75−95_ subgroup. The percentages of patients accepting DAPT, statin, β receptor blocker, ACEI or ARB treatment were comparable between PPCI-only and thrombus aspiration groups except for the use of DAPT in the G_76−95_ group (96.6 vs. 98.5%, *P* = 0.027). The utilization of anticoagulation therapy including GP IIb/IIIa inhibitor and low molecular heparin increased in the thrombus aspiration group (*P* < 0.05). The hospitalization day was prolonged with the increase of age and remained comparable between PPCI-only and thrombus aspiration groups (Table 2).

**Table 2:** The culprit lesion and in-hospital medication (*n* = 13,655).

	**G**_**21−50**_			**G**_**51−75**_			**G**_**76−95**_		
	**PPCI-only** **(*n =* 1,826)**	**PPCI+TA** **(*n =* 1,009)**	***P*** **-value**	**PPCI-only** **(*n =* 6,164)**	**PPCI+TA** **(*n =* 2,933)**	***P*** **-value**	**PPCI-only** **(*n =* 1,251)**	**PPCI+TA** **(*n =* 472)**	***P*** **-value**
**CULPRIT LESION**									
LM	27 (1.5%)	10 (1.0%)	0.273	103 (1.7%)	53 (1.8%)	0.640	29 (2.3%)	8 (1.7%)	0.426
LAD	1,084 (59.4%)	553 (54.8%)	**0.019**	3,470 (56.3%)	1,477 (50.4%)	**<0.001**	679 (54.3%)	207 (43.9%)	**<0.001**
LCX	307 (16.8%)	131 (13.0%)	**0.007**	999 (16.2%)	401 (13.7%)	**0.002**	183 (14.6%)	69 (14.6%)	0.996
RCA	629 (34.4%)	370 (36.7%)	0.235	2,377 (38.6%)	1,325 (45.2%)	**<0.001**	555 (44.4%)	253 (53.6%)	**0.001**
**IN-HOSPITAL MEDICATION**									
DAPT	1,788 (97.9%)	998 (98.9%)	0.053	6,042 (98.0%)	2,862 (97.6%)	0.172	1,209 (96.6%)	465 (98.5%)	**0.027**
Statin	1,752 (95.9%)	970 (96.1%)	0.807	5,868 (95.2%)	2,802 (95.5%)	0.479	1,186 (94.8%)	453 (96.0%)	0.314
β Receptor Blocker	1,082 (59.3%)	590 (58.5%)	0.685	3,111 (50.5%)	1,472 (50.2%)	0.801	534 (42.7%)	205 (43.4%)	0.780
ACE I or ARB	911 (49.9%)	481 (47.7%)	0.258	2,770 (44.9%)	1,336 (45.6%)	0.583	543 (43.4%)	197 (41.7%)	0.533
Glycoprotein IIb/IIIa inhibitor	1,093 (59.9%)	698 (69.2%)	**<0.001**	3,487 (56.6%)	1,877 (64.0%)	**<0.001**	538 (43.0%)	254 (53.8%)	**<0.001**
Low molecular heparin	1,355 (74.2%)	785 (77.8%)	**0.033**	4,454 (72.3%)	2,192 (74.7%)	**0.013**	797 (63.7%)	350 (74.2%)	**<0.001**
Hospitalization day	9 (7, 11)	9 (7, 11)	0.506	9 (7, 12)	9 (7, 12)	0.391	10 (8, 13)	10 (8, 13)	0.086

*Results are reported as mean ± SD, medians (25th−75th percentiles) or n (%), as appropriate. PPCI, primary percutaneous coronary intervention; TA, thrombus aspiration; LM, left main coronary artery; LAD, left anterior descending coronary artery; LCX, left circumflex coronary artery; RCA, right coronary artery; DAPT, dual antiplatelet therapy; ACEI, angiotensin-converting enzyme inhibitor; ARB, angiotensin receptor blocker. The bold values indicate that the values smaller than 0.5 implying significant difference*.

### Propensity Score Matching for Patients

After 1:1 propensity score matching for PPCI-only and thrombus aspiration groups, a total of 8,815 matched patients were obtained for the subsequent analysis. The detailed information was presented in Supplementary Tables 1, 2. In the G_21−50_ subgroup, only age (42.9 ± 5.3 years *vs*. 43.5 ± 5.8 years) and the utilization of DAPT (97.7 vs. 98.7%) were not well-matched (both *P* < 0.001). In the G_51−75_ subgroup, age and Killip class turned out to be comparable after matching. However, because of the possible complexity of characteristics presented in the G_51−75_ group, there were still differences in blood pressure, culprit lesion, and the utilization of DAPT, statin and anticoagulants. The matching worked excellently in the G_76−95_ subgroup, in which no significant difference of parameters remained between PPCI-only and thrombus aspiration groups.

### In-Hospital Clinical Outcomes

**Figure 3:**
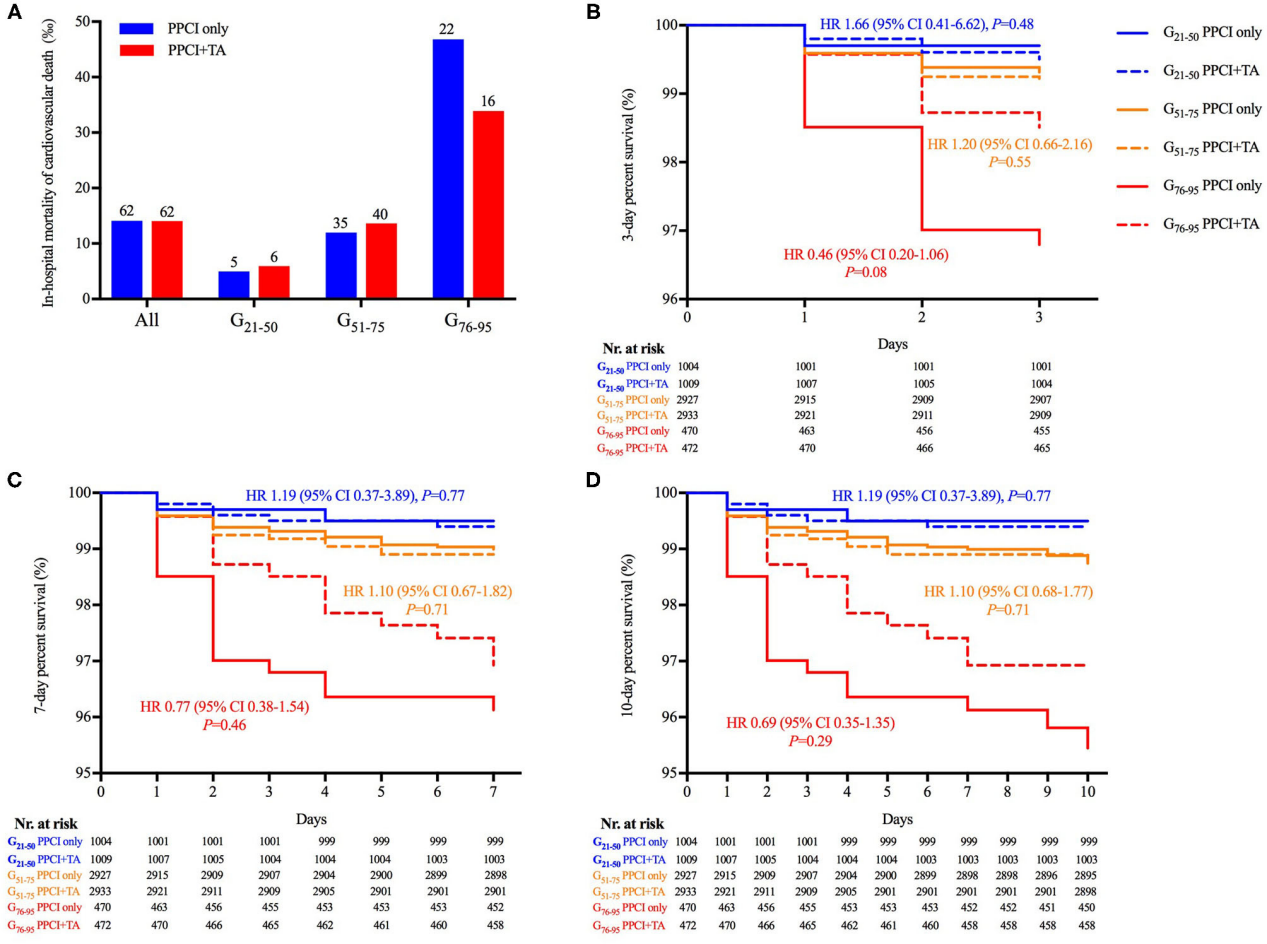
The in-hospital mortality and Kaplan–Meier curves of cardiovascular death. **(A)** The in-hospital mortality of cardiovascular death was comparable between PPCI-only and thrombus aspiration groups (*P* > 0.05). **(B–D)** Kaplan–Meier curve revealed that the in-hospital occurrence of cardiovascular death was comparable between PPCI-only and thrombus aspiration groups in each age subgroup. However, thrombus aspiration presented a tendency to decrease cardiovascular death at 3 days in the G_76−95_ subgroup (log-rank *P* = 0.08).

**Figure 4:**
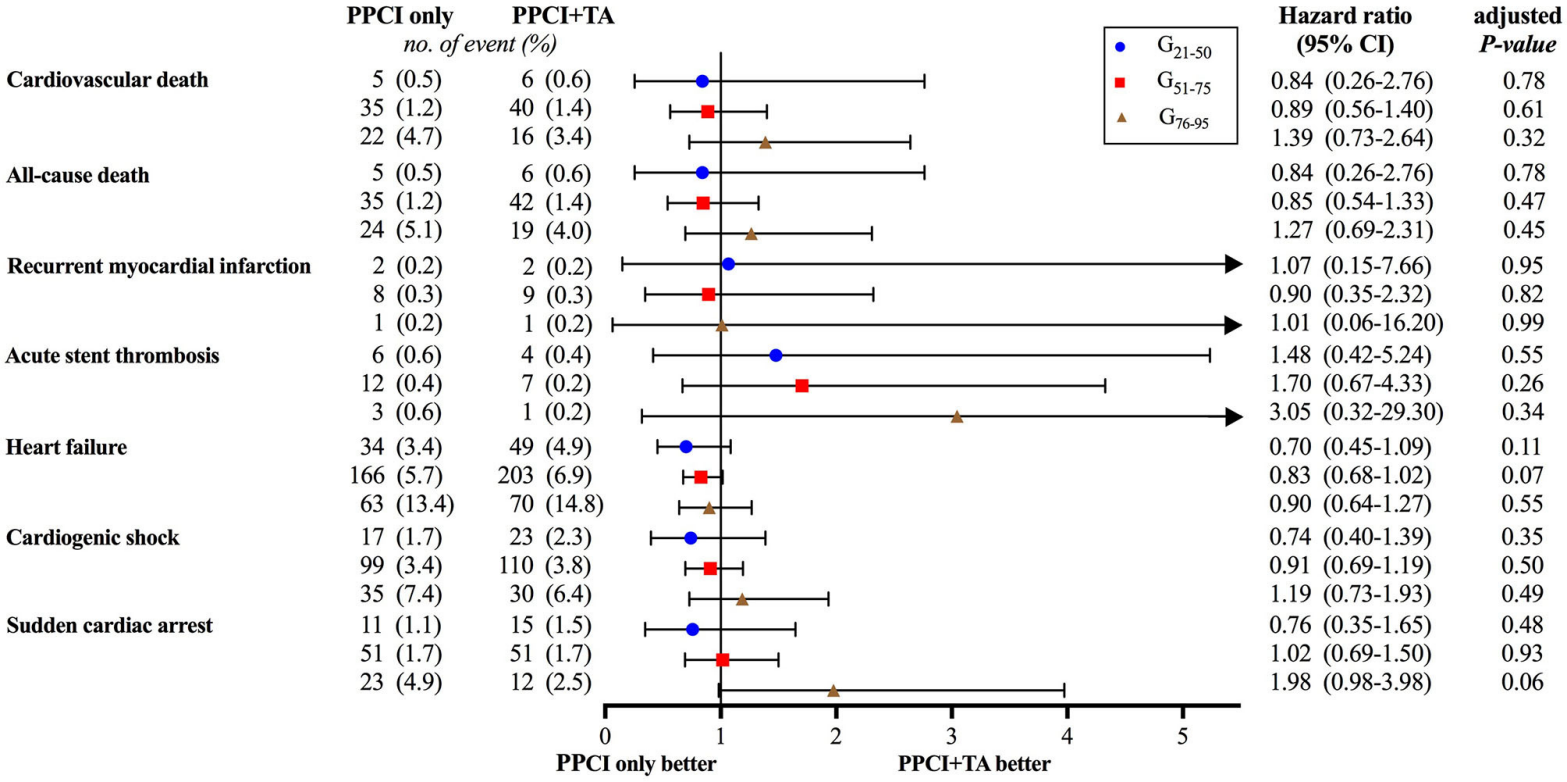
Forest plot of in-hospital adverse cardiovascular events. No significant differences were observed between PPCI-only and thrombus aspiration groups after adjustment for age.

The occurrence of in-hospital adverse cardiovascular events among the matched population was analyzed. The rates of the primary outcome, namely in-hospital cardiovascular death, were 0.5, 1.3 and 4.0% in each age subgroup, showing no significant difference between PPCI-only and thrombus aspiration groups. Nevertheless, thrombus aspiration reduced the occurrence of cardiovascular death by 27.7% (Figure 3A). Although the rates of cardiovascular death at 3, 7, and 10 days were comparable between PPCI-only and thrombus aspiration groups in all age subgroups (Figures 3B–D), thrombus aspiration presented the tendency to reduce the occurrence of in-hospital cardiovascular death at 3 days (HR 0.46; 95% CI, 0.20–1.06; log-rank *P* = 0.08). Additionally, the rates of secondary outcomes including all-cause death, recurrent myocardial infarction, acute stent thrombosis, heart failure, cardiogenic shock, sudden cardiac arrest and stroke were comparable between two treatment groups in three age subgroups (all *P* > 0.05 after adjustment for age, Figure 4). While, in the G_51−75_ group, we observed a marginally higher rate of heart failure in patients undergoing thrombus aspiration (5.7 vs. 6.9%, adjusted *P* = 0.07). Meanwhile, the rate of sudden cardiac arrest was insignificantly lower in the thrombus aspiration group (4.9 vs. 2.5%, adjusted *P* = 0.06) for the elderly.

**Figure 5:**
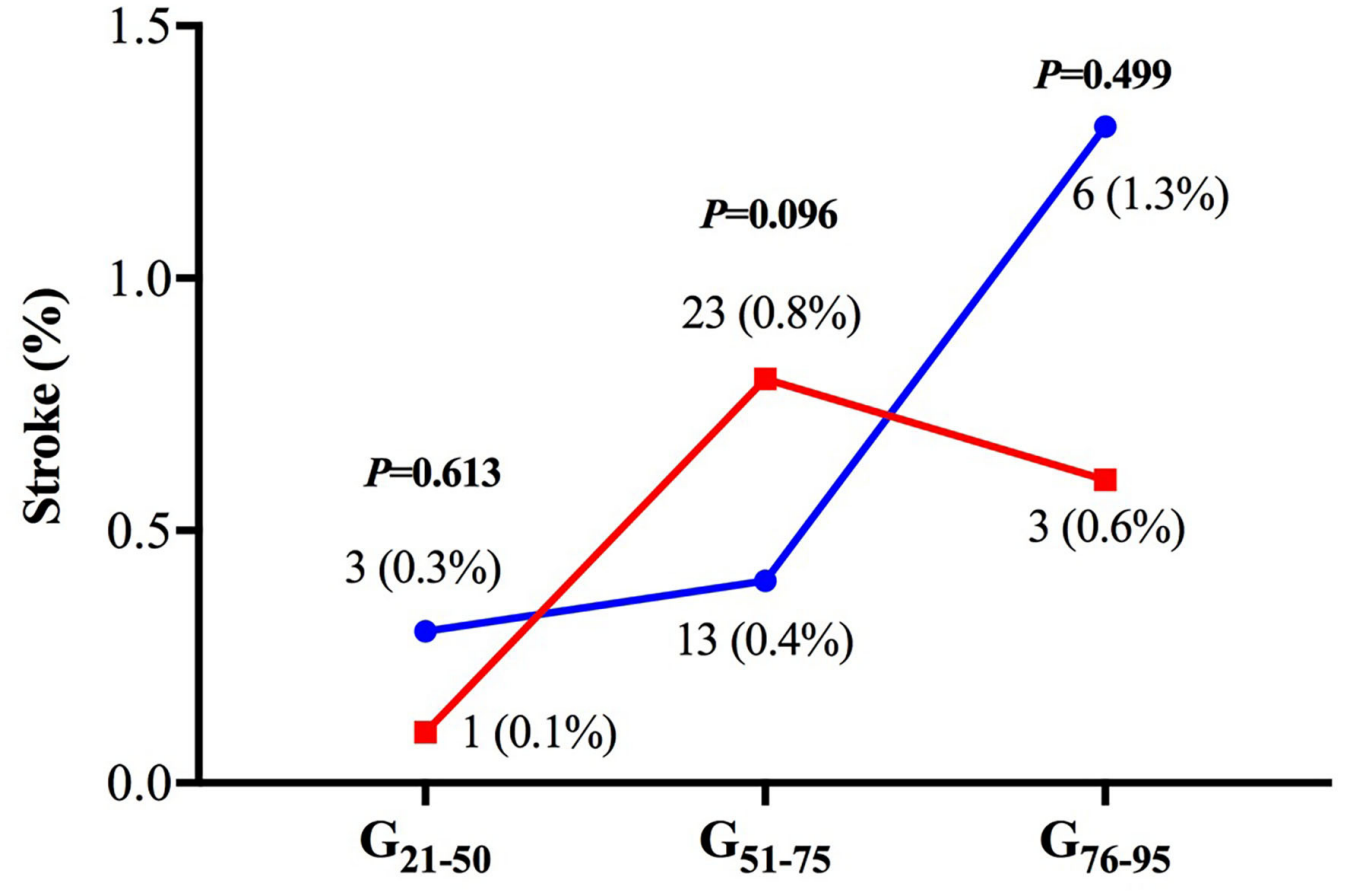
The incidence of in-hospital stroke. There was no significant difference in the risk of in-hospital stroke between the two treatment groups in all age subgroups.

Stroke was regarded as the key safety outcome, of which the incidence increased with aging and sustained the similarity between PPCI-only and thrombus aspiration groups (all *P* > 0.05, Figure 5).

The last echocardiography examination before discharge presented that among patients ≤ 75 years old, LVEF was lower and the rate of regional wall motion abnormality was higher in the thrombus aspiration group (both *P* < 0.05), while no significant difference was observed in the G_76−95_ subgroup. Also, the incidence of regional wall motion abnormality raised with the increase of age (Supplementary Figure 2).

## Discussion

To the best of our knowledge, it is the largest nationwide, multicenter, retrospective epidemiological study to evaluate the age-associated utilization and clinical outcomes of thrombus aspiration among STEMI patients undergoing PPCI within 12 h of symptoms onset and implanting DES in China. We demonstrated that thrombus aspiration did not increase the rate of in-hospital major adverse cardiac cerebrovascular events (e.g., cardiovascular death and stroke). However, the utilization of thrombus aspiration in patients older than 75 years helped maintain LVEF and regional wall motion normality and presented the tendency toward diminishing the risk of in-hospital cardiovascular death at 3 days and sudden cardiac arrest.

According to the data from the CCC–ACS database, thrombus aspiration is still being used in a substantial number of STEMI patients. A 32.3% the STEMI patients undergoing PPCI and implanting DES were treated with upfront or bailout thrombus aspiration. STEMI patients were less likely to be treated with thrombus aspiration with the increase of age. We found that the most frequently encountered risk factor of STEMI was smoking in patients ≤ 75 years and hypertension in patients >75 years, this phenomenon was partially in line with the previous findings (18, 19). For patients aged 21–50 years, the percentage of smokers was higher in the thrombus aspiration group.

In the entire population, the incidences of in-hospital adverse cardiovascular events were comparable between PPCI-only and thrombus aspiration groups. This finding was sort of consistent with the results in TASTE, TOTAL and a large observational study (*n* = 42,829) using available data from the Swedish Coronary Angiography and Angioplasty Registry (SCAAR) at 30 days (7, 8, 20). Due to the ventricular remodeling, diastolic dysfunction and decreased vascular compliance with aging, the elderly presented higher mortality when suffering from the sudden occlusion of the coronary artery (21). Nevertheless, we detected that thrombus aspiration tended to reduce the risk of in-hospital cardiovascular death at 3 days by 27.6% in the G_76−95_ group. It is noteworthy that thrombus aspiration also tended to decline the rate of rehospitalization due to reinfarction (0.5 vs. 0.9%; HR 0.61; 95% CI, 0.34–1.07; *P* = 0.09) and stent thrombosis (0.2 vs. 0.5%; HR 0.47; 95% CI, 0.20–1.02; *P* = 0.06) at 30 days in TASTE (7). Meanwhile, we noticed that the incidence of stent thrombosis in the PPCI-only group was 1.5–3 times compared to that in the thrombus aspiration group, although the difference was insignificant. Considering the extremely low incidence of stent thrombosis, whether thrombus aspiration can reduce the occurrence of this event needs to be further investigated in a larger population. What is more, thrombus aspiration showed a protective impact on reducing the prevalence of sudden cardiac death in the elderly (4.9 vs. 2.5%, *P* = 0.06). While, in the G_51−75_ group, we observed a higher rate of heart failure in patients undergoing thrombus aspiration, which may raise physicians' attention in the clinical practice.

A part of the information derived from angiography and the last echocardiography before discharge was recorded in the CCC–ACS database. Among patients ≤ 75 years, the culprit lesion suffered from thrombus aspiration was mainly located in LAD, LVEF was lower and the rate of regional wall motion abnormality was higher in the thrombus aspiration group. STEMI patients with a high thrombus burden are more likely to benefit from thrombus aspiration compared with patients with a low thrombus burden, suggesting the more frequent utilization of thrombus aspiration in patients with high thrombus burden (10). Ahmed et al. uncovered that the higher thrombus grade was associated with larger infarct size and slightly worse LV function (22). Hence, we hypothesized that the deterioration of cardiac function and myocardial wall motion might be associated with the burden of coronary thrombus rather than the treatment. Unfortunately, the severe impairment of cardiac performance cannot be simply renovated by thrombus aspiration in a short time. By contrast, among patients > 75 years, the culprit lesion suffered from thrombus aspiration was mainly located in RCA, and LVEF and the incidence of regional wall motion abnormality were comparable between the two treatment groups. Unlike LAD predominantly supplying blood to the anterior and septal proportions of LV myocardium, RCA principally supplies the right ventricle and atrium (23). Meanwhile, the elderly patients suffering from persistent angina have robust collateral circulation to restore flow, which might help remain the blood supply and cardiac function to some extent (24).

There are still debates on whether thrombus aspiration can increase the rate of stroke. In this study, we did not find significantly increased in-hospital occurrence of the stroke in the thrombus aspiration group, which was similar in TASTE and other research studies (7, 20). Although TOTAL propagated the attitude that thrombus aspiration could increase the occurrence of a stroke at 30 and 180 days (*P* = 0.020 and 0.002, respectively), the conclusion was drawn among the randomized population with crossover from their initial treatment allocation to the alternative therapy in both groups. Looking back on the on-treatment analysis among patients who received upfront or bailout thrombus aspiration irrespective of randomization and convention PCI in TOTAL, the thrombus aspiration could not increase the rate of stroke at 30 and 180 days (*P* = 0.193 and 0.068, respectively) (8). TOTAL also indicated that the application of thrombus aspiration among patients with high thrombus burden (thrombolysis in myocardial infarction [TIMI] thrombus Grade ≥3) rather than those with low thrombus burden could increase the rate of stroke at 30 days (0.7% in thrombus aspiration group *vs*. 0.4% in PCI-only group; HR = 1.90; 95% CI, 1.04–3.08; *P* = 0.03) (25). In the TOTAL trial, the minimal volume of thrombus aspiration procedures required for operators only reached five in the last 2 years (14). The utilization of upfront bailout GP IIb/IIIa inhibitor was more frequent among the patients undergoing thrombus aspiration treatment than those in the PCI-only group (25). Hence, we cannot rule out that the higher rate of stroke was caused by procedures during thrombus aspiration therapy or more intensive anticoagulation therapy after thrombus aspiration. It is a consensus that the inappropriate operation of thrombus aspiration devices may cause systemic embolization of thrombotic material or air embolism (26). So, the development of aspiration catheters and improvement of thrombus aspiration skills need to be emphasized to avoid the increased ischemic stroke risk, especially among patients with a high thrombus burden. Meanwhile, the patients with a high thrombus burden are more likely to suffer from hemorrhagic stroke due to the intensive anticoagulants and need to be closely monitored in intensive or cardiac care units post procedurally.

## Limitations

There were several limitations in this study. First, the CCC–ACS project is a retrospective and observational real-world study based on the medical records uploaded in the database, limited data were gathered. Hence, the detailed information of properties of thrombus (red/white), location of thrombus (proximal/distal), TIMI thrombus grade, size and length of the stent, bifurcation, collateral circulation and so on were inaccessible. However, this study still provides physicians with a better understanding of the utilization and clinical benefit of thrombus aspiration. Second, due to the numerous factors considered, there were still differences with the potential confounders between PPCI-only and thrombus aspiration groups after 1:1 propensity score matching. While, most variables were well-matched, the difference of age in the unmatched population was taken into consideration during the analysis of clinical outcomes to increase the reliability of results.

## Conclusion

In this large nationwide observational study, thrombus aspiration did not significantly reduce the in-hospital risk of cardiovascular death, all-cause death, recurrent myocardial infarction, acute stent thrombosis, heart failure, cardiogenic shock, sudden cardiac arrest and stroke compared with conventional PPCI among the whole STEMI patients. However, it presented the tendency to reduce the occurrence of in-hospital cardiovascular death at 3 days and sudden cardiac arrest among patients older than 75 years, and increase the risk of heart failure in patients aged 51–75 years.

## Data Availability Statement

The datasets presented in this article are not readily available because the datasets analyzed in this study are not publicly available according to the regulation of CCC-ACS project, but the reliability and accuracy can be validated with the assistance of Prof. Dong Zhao who is the principle investigator of this project. Requests to access the datasets should be directed to Prof. Dong Zhao, deezhao@vip.sina.com.

## Ethics Statement

The studies involving human participants were reviewed and approved by the Beijing Anzhen Hospital. Written informed consent for participation was not required for this study in accordance with the national legislation and the institutional requirements.

## Author Contributions

G-SM and L-JC contributed to the design and overall investigation. Y-YQ was responsible for the data collection, statistical analysis, and manuscript. X-GZ, C-WJ, Y-MS, RZ, W-JZ, and Z-JJ have made substantial contributions to the analysis and interpretation of data or revising the manuscript. All authors read and approved the final manuscript.

## Funding

CCC–ACS project was a collaborative study co-launched by the American Heart Association (AHA) and the Chinese Society of Cardiology (CSC). The AHA was funded by Pfizer and AstraZeneca for the quality improvement initiative through an independent grant for learning and change.

## Conflict of Interest

The authors declare that the research was conducted in the absence of any commercial or financial relationships that could be construed as a potential conflict of interest.

## Publisher's Note

All claims expressed in this article are solely those of the authors and do not necessarily represent those of their affiliated organizations, or those of the publisher, the editors and the reviewers. Any product that may be evaluated in this article, or claim that may be made by its manufacturer, is not guaranteed or endorsed by the publisher.

## Supplementary Material

Supplementary Figure 1 Logistic regression analysis revealed that thrombus aspiration was less likely to be conducted with the increase of age. PPCI, primary percutaneous coronary intervention; TA, thrombus aspiration; CI, confidence interval.

Supplementary Figure 2 LVEF and the rate of region wall motion abnormality before discharge. **(A,B)** For patients ≤ 75 years, patients undergoing thrombus aspiration treatment presented with lower LVEF and a higher rate of regional wall motion abnormality. While for patients > 75 years, no significant difference was observed between the two treatment groups. RWMA, regional wall motion abnormality.

Supplementary Table 1 The baseline characteristics of the matched patients (*n* = 8,815).

Supplementary Table 2 The culprit lesion and in-hospital medication of the matched patients (*n* = 8,815).

Supplementary Table 3 One hundred and fifty hospitals and investigators participating in the CCC-ACS project during 2014–2017.

## Abbreviations

ACEI: Angiotensin-converting enzyme inhibitor
AHA: American Heart Association
ARB: Angiotensin receptor blocker
CCC-ACS project: The Improving Care for Cardiovascular Disease in China-Acute Coronary Syndrome project
CI: Confidence interval
CSC: Chinese Society of Cardiology
DAPT: Dual antiplatelet therapy
DES: Drug-eluting stent
GP IIb/IIIa inhibitor: Glycoprotein IIb/IIIa inhibitor
HR: Hazard ratio
LAD: Left anterior descending branch
LCX: Left circumflex branch
LM: Left main coronary artery
LVEF: Left-ventricular ejection fraction
OR: Odds ratio
PCI: Percutaneous coronary intervention
PPCI: Primary percutaneous coronary intervention
RCA: Right coronary artery
SBP: Systolic blood pressure
SD: Standard deviation
STEMI: ST-segment elevation myocardial infarction
TAPAS: The Thrombus Aspiration during Percutaneous Coronary Intervention in Acute Myocardial Infarction Study
TASTE: The Thrombus Aspiration in ST-Elevation Myocardial Infarction in Scandinavia
TOTAL: Trial of Routine Aspiration Thrombectomy with PCI vs. PCI Alone in Patients with STEMI.
