# Supplementary figures and images for: Age-Related Utilization of Thrombus Aspiration in Patients With ST-Segment Elevation Myocardial Infarction: Findings From the Improving Care for Cardiovascular Disease in China Project
Source: Front Cardiovasc Med. 2022 Feb 21;9:791007. doi: 10.3389/fcvm.2022.791007 (PMC8898949; doi:10.3389/fcvm.2022.791007)

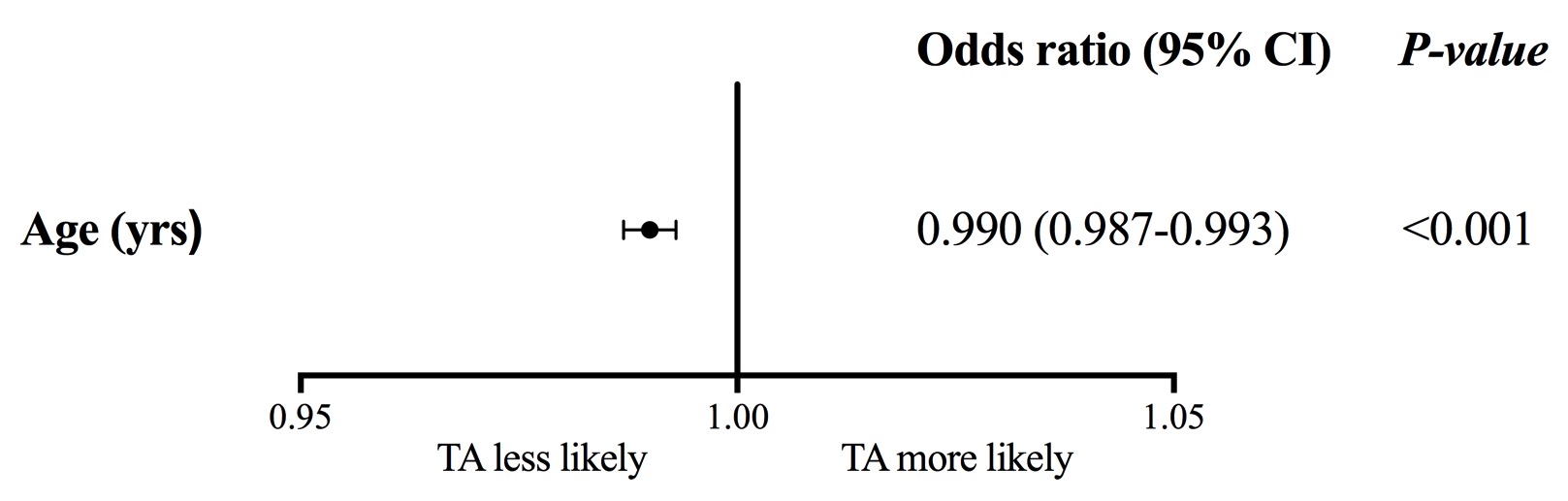

Supplement: Supplementary Figure 1 — Logistic regression analysis revealed that thrombus aspiration was less likely to be conducted with the increase of age. PPCI, primary percutaneous coronary intervention; TA, thrombus aspiration; CI, confidence interval. [file Image_1.JPEG]

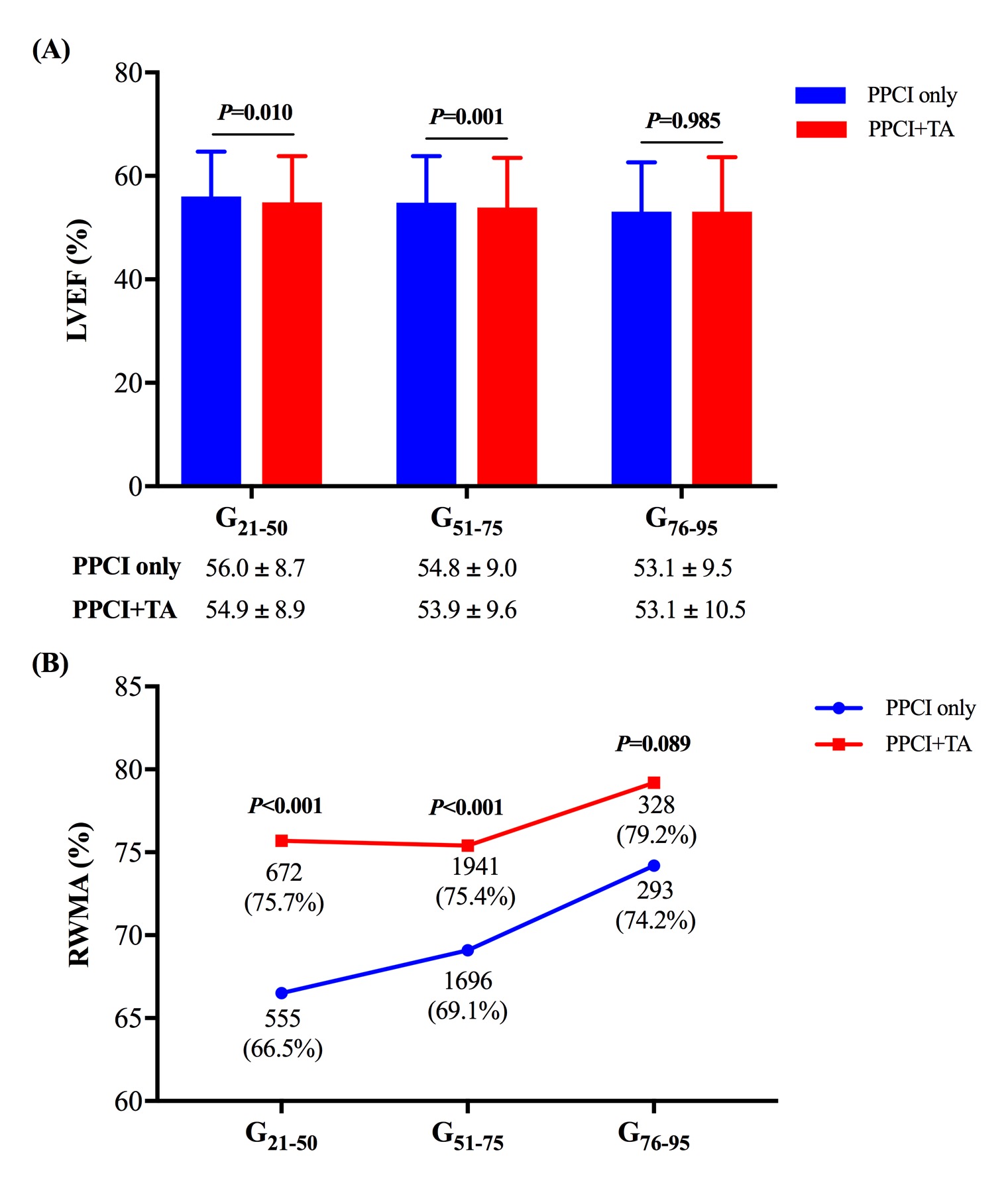

Supplement: Supplementary Figure 2 — LVEF and the rate of region wall motion abnormality before discharge. (A,B) For patients ≤ 75 years, patients undergoing thrombus aspiration treatment presented with lower LVEF and a higher rate of regional wall motion abnormality. While for patients > 75 years, no significant difference was observed between the two treatment groups. RWMA, regional wall motion abnormality. [file Image_2.JPEG]
