# Supplementary material for: Age-Related Utilization of Thrombus Aspiration in Patients With ST-Segment Elevation Myocardial Infarction: Findings From the Improving Care for Cardiovascular Disease in China Project
Source: Front Cardiovasc Med. 2022 Feb 21;9:791007. doi: 10.3389/fcvm.2022.791007 (PMC8898949; doi:10.3389/fcvm.2022.791007)
Supplement: Supplementary Table 1 — The baseline characteristics of the matched patients (n = 8,815). [file Data_Sheet_1.pdf]

Supplementary Table S1. The baseline characteristics of the matched patients (n=8815).

	G_{21-50}			G_{51-75}			G_{76-95}		
	**PPCI-only (n=1004)**	**PPCI+TA (n=1009)**	***P*-value**	**PPCI-only (n=2927)**	**PPCI+TA (n=2933)**	***P*-value**	**PPCI-only (n=470)**	**PPCI+TA (n=472)**	***P*-value**
**Baseline information**									
Age (years)	42.9±5.3	43.5±5.8	**<0.001**	61.7±6.4	62.0±6.8	0.188	80.4±3.6	80.2±3.7	0.290
Male, n (%)	966 (96.2%)	970 (96.1%)	0.925	2382 (81.4%)	2398 (81.8%)	0.708	282 (60.0%)	295 (62.5%)	0.431
BMI (kg/m^2)	25.4±3.4	25.2±3.4	0.576	24.5±3.0	24.5±2.9	0.938	23.7±3.3	23.5±3.1	0.758
Heart Rate (bpm)	80.9±15.1	81.2±15.2	0.498	77.3±15.8	76.8±15.7	0.328	76.1±18.2	76.2±18.0	0.728
SBP (mmHg)	128.0±22.3	127.0±22.6	0.285	126.4±23.6	123.9±23.1	**<0.001**	122.7±24.5	123.6±24.1	0.540
DBP (mmHg)	81.7±15.7	80.9±16.0	0.185	77.6±14.9	76.2±14.6	**<0.001**	71.3±14.9	72.3±13.8	0.228
Killip Class									
Ⅰ, n (%)	829 (82.6%)	843 (83.5%)	0.558	2286 (78.1%)	2329 (79.4%)	0.222	339 (72.1%)	327 (69.3%)	0.337
Ⅱ, n (%)	140 (13.9%)	129 (12.8%)	0.445	485 (16.6%)	437 (14.9%)	0.079	82 (17.4%)	105 (22.2%)	0.065
Ⅲ, n (%)	8 (0.8%)	13 (1.3%)	0.278	44 (1.5%)	49 (1.7%)	0.608	10 (2.1%)	15 (3.2%)	0.316
Ⅳ, n (%)	27 (2.7%)	24 (2.4%)	0.657	112 (3.8%)	118 (4.0%)	0.698	39 (8.3%)	25 (5.3%)	0.067
**Medical History**									
Smoking	713 (71.0%)	724 (71.8%)	0.714	1577 (53.9%)	1542 (52.6%)	0.317	121 (25.7%)	130 (27.5%)	0.533
Prior Myocardial Infarction	29 (2.9%)	25 (2.5%)	0.568	138 (4.7%)	136 (4.6%)	0.888	18 (3.8%)	19 (4.0%)	0.877
Prior PCI	27 (2.7%)	26 (2.6%)	0.875	147 (5.0%)	143 (4.9%)	0.796	23 (4.9%)	23 (4.9%)	0.988
Hypertension	365 (36.4%)	378 (37.5%)	0.606	1436 (49.1%)	1474 (50.3%)	0.360	263 (56.0%)	267 (56.6%)	0.850
Hyperlipemia	84 (8.4%)	87 (8.6%)	0.837	195 (6.7%)	212 (7.2%)	0.394	34 (7.2%)	27 (5.7%)	0.345
Diabetes Mellitus	124 (12.4%)	136 (13.5%)	0.451	628 (21.5%)	599 (20.4%)	0.331	86 (18.3%)	88 (18.6%)	0.891
Stroke	16 (1.6%)	20 (2.0%)	0.511	245 (8.4%)	258 (8.8%)	0.560	50 (10.6%)	61 (12.9%)	0.277

Results are reported as mean ± SD or n (%). PPCI, primary percutaneous coronary intervention; TA, thrombus aspiration; BMI, body mass index; SBP, systolic blood pressure; DBP, diastolic blood pressure.

Supplementary Table S2. The culprit lesion and in-hospital medication of the matched patients (n=8815).

	G_{21-50}			G_{51-75}			G_{76-95}		
	**PPCI-only (n=1004)**	**PPCI+TA (n=1009)**	***P*-value**	**PPCI-only (n=2927)**	**PPCI+TA (n=2933)**	***P*-value**	**PPCI-only (n=470)**	**PPCI+TA (n=472)**	***P*-value**
**Culprit lesion**									
LM	10 (1.0%)	10 (1.0%)	0.991	48 (1.6%)	53 (1.8%)	0.623	8 (1.7%)	8 (1.7%)	0.993
LAD	582 (58.0%)	553 (54.8%)	0.153	1608 (54.9%)	1477 (50.4%)	**<0.001**	219 (46.6%)	207 (43.9%)	0.398
LCX	150 (14.9%)	131 (13.0%)	0.177	467 (16.0%)	401 (13.7%)	**0.014**	76 (16.2%)	69 (14.6%)	0.509
RCA	329 (32.8%)	370 (36.7%)	0.066	1189 (40.6%)	1325 (45.2%)	**<0.001**	246 (52.3%)	253 (53.6%)	0.698
**Medication in hospital**									
DAPT	981 (97.7%)	998 (98.9%)	**0.037**	2827 (96.6%)	2862 (97.6%)	**0.024**	457 (97.2%)	465 (98.5%)	0.172
Statin	958 (95.4%)	970 (96.1%)	0.424	2762 (94.4%)	2802 (95.5%)	**0.041**	444 (94.5%)	453 (96.0%)	0.278
β Receptor Blocker	601 (59.9%)	590 (58.5%)	0.527	1439 (49.2%)	1472 (50.2%)	0.433	185 (39.4%)	205 (43.4%)	0.205
ACE I or ARB	487 (48.5%)	481 (47.7%)	0.708	1340 (45.8%)	1336 (45.6%)	0.860	180 (38.3%)	197 (41.7%)	0.281
Glycoprotein IIb/IIIa inhibitor	680 (67.7%)	698 (69.2%)	0.484	1714 (58.6%)	1877 (64.0%)	**<0.001**	256 (54.5%)	254 (53.8%)	0.840
Low Molecular Heparin	768 (76.5%)	785 (77.8%)	0.485	2115 (72.3%)	2192 (74.7%)	**0.032**	331 (70.4%)	350 (74.2%)	0.201
**Hospitalization Day**	9 (7, 11)	9 (7, 11)	0.605	9 (8, 12)	9 (7, 12)	0.091	10 (8, 14)	10 (8, 13)	0.787

Results are reported as mean ± SD, medians (25th–75th percentiles) or n (%), as appropriate. PPCI, primary percutaneous coronary intervention; TA, thrombus aspiration; LM, left main coronary artery; LAD, left anterior descending coronary artery; LCX, left circumflex coronary artery; RCA, right coronary artery; DAPT, dual antiplatelet therapy; ACEI, angiotensin-converting enzyme inhibitor; ARB, angiotensin receptor blocker.

Supplementary Table S3. One hundred and fifty hospitals and investigators participating in the CCC-ACS project during 2014-2017.

Hospitals	Province	City	Investigator
Shanxi Cardiovascular Hospital	Shanxi	Taiyuan	Bao Li
Nanjing Drum Tower Hospital, The Affiliated Hospital of Nanjing University Medical School	Jiangsu	Nanjing	Biao Xu<br>Guangshu Han
Hainan General Hospital	Hainan	Haikou	Bin Li
The Second Hospital of Jilin University	Jilin	Changchun	Bin Liu
The 2nd Affiliated Hospital of Harbin Medical University	Heilongjiang	Harbin	Bo Yu
The Ninth Hospital Affiliated to Shanghai Jiaotong University School of Medicine	Shanghai	Shanghai	Changqian Wang
Henan Provincial People's Hospital	Henan	Zhengzhou	Chuanyu Gao
Shanxi Provincial People's Hospital	Shanxi	Taiyuan	Chunlin Lai
Xinqiao Hospital, Third Military Medical University	Chongqing	Chongqing	Cui Bin<br>Lan Huang
China Meitan General Hospital	Beijing	Beijing	Di Wu
The 309th Hospital of Chinese People's Liberation Army	Beijing	Beijing	Fakuan Tang<br>Jun Xiao
Zhongda Hospital, Southeast University	Jiangsu	Nanjing	Genshan Ma
The First Affiliated Hospital of Liaoning Medical University	Liaoning	Jinzhou	Guizhou Tao
Xinjiang Uygur Autonomous Region People's Hospital	Xinjiang	Urumchi	Guoqing Li
Sir Run Run Shaw Hospital, College of Medicine, Zhejiang University	Zhejiang	Hangzhou	Guosheng Fu
Beijing Friendship Hospital, Capital Medical University	Beijing	Beijing	Hongwei Li
The First Affiliated Hospital of Bengbu Medical College	Anhui	Bengbu	Honhju Wang
General Hospital of TISCO	Shanxi	Taiyuan	Huifeng Wang
Dongguan People's Hospital	Guangdong	Dongguan	Jianfeng Ye
Panyu Hospital of Chinese Medicine	Guangdong	Guangzhou	Jianhao Li
Peking University First Hospital	Beijing	Beijing	Jie Jiang
Sun Yat-sen Memorial Hospital, Sun Yat-sen University	Guangdong	Guangzhou	Jingfeng Wang
Guangdong General Hospital	Guangdong	Guangzhou	Jiyan Chen
Hospital of Xinjiang Production & Construction Corps	Xinjiang	Urumchi	Junming Liu
The Military General Hospital of Beijing PLA	Beijing	Beijing	Junxia Li
The First Affiliated Hospital of Guangxi Medical University	Guangxi	Nanning	Lang Li
Tongren Hospital Affiliated to Shanghai Jiaotong	Shanghai	Shanghai	Li Jiang
**Hospitals**	**Province**	**City**	**Investigator**
University School of Medicine			
Binzou City Center Hospital	Shandong	Binzhou	Lijun Meng
The First Affiliated Hospital of Zhengzhou University	Henan	Zhengzhou	Ling Li
Xijing Hospital	Shaanxi	Xi'an	Ling Tao
The Affiliated Hospital of Guizhou Medical University	Guizhou	Guiyang	Lirong Wu
First Affiliated Hospital of the People's Liberation Army General Hospital	Beijing	Beijing	Miao Tian
The Second People's Hospital of Yunnan Province	Yunnan	Kunming	Minghua Han
Haikou People's Hospital	Hainan	Haikou	Moshui Chen
Gansu Provincial Hospital	Gansu	Lanzhou	Ping Xie
The First Affiliated Hospital of Henan University of Science and Technology	Henan	Luoyang	Pingshuan Dong
Chenzhou First People's Hospital	Hunan	Chenzhou	Qiaoqing Zhong

People’s Hospital of Qinghai Province	Qinghai	Xining	Rong Chang
Affiliated Hospital of Ningxia Medical University	Ningxia	Yinchuan	Shaobin Jia
Beijing Anzhen Hospital, Capital Medical University	Beijing	Beijing	Shaoping Nie<br>Xiaohui Liu
North Jiangsu People's Hospital	Jiangsu	Yangzhou	Shenghu He
Shanghai Sixth People's Hospital	Shanghai	Shanghai	Shixin Ma
The First Hospital of Handan	Hebei	Handan	Shuanli Xin
Huai'an First People's Hospital	Jiangsu	Huai'an	Shuren Ma
The First Affiliated Hospital of Chongqing Medical University	Chongqing	Chongqing	Suxin Luo
Navy General Hospital	Beijing	Beijing	Tianchang Li
Zhejiang Provincial Hospital of TCM	Zhejiang	Hangzhou	Wei Mao
The Third Xiangya Hospital of Central South University	Hunan	Changsha	Weihong Jiang
Affiliated Hospital of Qinghai University	Qinghai	Xining	Weijun Liu
Teda International Cardiovascular Hospital	Tianjin	Tianjin	Wenhua Lin
The Second Hospital of Hebei Medical University	Hebei	Shijiazhuang	Xianghua Fu
Changhai Hospital of Shanghai	Shanghai	Shanghai	Xianxian Zhao
The Second Affiliated Hospital to Nanchang University	Jiangxi	Nanchang	Xiaoshu Cheng
Hebei General Hospital	Hebei	Shijiazhuang	Xiaoyong Qi
Inner Mongolia People's Hospital	Inner Mongolia	Hohhot	Xingsheng Zhao
The General Hospital of Shenyang Military Region	Liaoning	Shenyang	Yaling Han
The First Hospital of Jilin University	Jilin	Changchun	Yang Zheng
**Hospitals**	**Province**	**City**	**Investigator**
Tianjin Chest Hospital	Tianjin	Tianjin	Yin Liu
Hunan Provincial People's Hospital	Hunan	Changsha	Ying Guo
People's Hospital of Yuxi City	Yunnan	Yuxi	Yinglu Hao
The People's Hospital of Guangxi Zhuang Autonomous Region	Guangxi	Nanning	Yingzhong Lin
The First Teaching Hospital of Xinjiang Medical University	Xinjiang	Urumchi	Yitong Ma
Baogang Hospital	Inner Mongolia	Baotou	Yongdong Li
Tianjin Medical University General Hospital	Tianjin	Tianjin	Yuemin Sun
The Second Affiliated Hospital of Zhengzhou University	Henan	Zhengzhou	Yulan Zhao
Nanfang Hospital of Southern Medical University	Guangdong	Guangzhou	Yuqing Hou
The First Affiliated Hospital to Nanchang University	Jiangxi	Nanchang	Zeqi Zheng
The First Affiliated Hospital of Lanzhou University	Gansu	Lanzhou	Zheng Zhang
The Third Hospital of Shijiazhuang	Hebei	Shijiazhuang	Zhenguo Ji
Wuxi People's Hospital	Jiangsu	Wuxi	Zhenyu Yang
Jiangsu Province Hospital	Jiangsu	Nanjing	Zhijian Yang
The Second Hospital of Shanxi Medical University	Shanxi	Taiyuan	Zhiming Yang
The Affiliated Hospital of Xuzhou Medical College	Jiangsu	Xuzhou	Zhirong Wang
Southwest Hospital, Third Military Medical University	Chongqing	Chongqing	Zhiyuan Song
The First Affiliated Hospital of Xi’an Jiaotong University	Shaanxi	Xi'an	Zuyi Yuan
Yangzhou First People's Hospital	Jiangsu	Yangzhou	Aihua Li
Hospital 463 of Chinese People's Liberation Army	Liaoning	Shenyang	Bosong Yang
The Central Hospital of Mianyang	Sichuan	Mianyang	Caidong Luo
Liaocheng People's Hospital	Shandong	Liaocheng	Chunyan Zhang
Yancheng Third People's Hospital	Jiangsu	Yancheng	Chunyang Wu

The Second Xiangya Hospital of Central South University	Hunan	Changsha	Daoquan Peng
The Central Hospital of Panzhihua	Sichuan	Panzhihua	Dawen Xu
The First Hospital of Qiqihaer City	Heilongjiang	Qiqihaer	Gang Xu
The Third the People's Hospital of Bengbu	Anhui	Bengbu	Gengsheng Sang
The First Hospital of Jiamusi	Heilongjiang	Jiamusi	Guixia Zhang
Zhoushan People's Hospital	Zhejiang	Zhoushan	Guoxiong Chen
Dalian Municipal Central Hospital	Liaoning	Dalian	Hailong Lin
Renmin Hospital of Wuhan University	Hubei	Wuhan	Hong Jiang
Ningxia People's Hospital	Ningxia	Yinchuan	Hong Luan
The First People's Hospital of Yunnan Province (Kunhua	Yunnan	Kunming	Hong Zhang
**Hospitals**	**Province**	**City**	**Investigator**
Hospital)			
The Central Hospital of Zhoukou	Henan	Zhoukou	Hualing Liu
Anyang District Hospital	Henan	Anyang	Hui Liu
Sichuan Provincial People's Hospital	Sichuan	Chengdu	Jianhong Tao
Mudanjiang Cardiovascular Disease Hospital	Heilongjiang	Mudanjiang	Jianwen Liu
Yichang Central Hospital	Hubei	Yichang	Jiawang Ding
Qilu Hospital of Shandong University	Shandong	Jinan	Jifu Li
Affiliated Hospital of Jiangsu University	Jiangsu	Zhenjiang	Jinchuan Yan
The First People's Hospital of Nanning City	Guangxi	Nanning	Jinru Wei
The First Affiliated Hospital of Fujian Medical University	Fujian	Fuzhou	Jinzi Su
Chengdu Third People's Hospital	Sichuan	Chengdu	Jiong Tang
Yantaishan hospital	Shandong	Yantai	Juexin Fan
Qingdao Municipal Hospital	Shandong	Qingdao	Jun Guan
Zhongshan Hospital Affiliated to Fudan University	Shanghai	Shanghai	Junbo Ge
Longyan First Hospital	Fujian	Longyan	Kaihong Chen
Affiliated Hospital of Guangdong Medical College	Guangdong	Guangzhou	Keng Wu
Jiangxi Provincial People's Hospital	Jiangxi	Nanchang	Lang Ji
Anhui Provincial Hospital	Anhui	Hefei	Likun Ma
Xiangtan City Central Hospital	Hunan	Xiangtan	Lilong Tang
The First Hospital of Haerbin City	Heilongjiang	Harbin	Lin Wei
Central Hospital Affiliated to Shenyang Medical College	Liaoning	Shenyang	Man Zhang<br>Kaiming Chen
The Central Hospital of Wuhan	Hubei	Wuhan	Manhua Chen
Hangzhou First People's Hospital	Zhejiang	Hangzhou	Ningfu Wang
The Central Hospital of Xuzhou	Jiangsu	Xuzhou	Peiying Zhang
The Second hospital of Dalian Medical University	Liaoning	Dalian	Peng Qu
The First Affiliated Hospital of Liaoning University of Traditional Chinese Medicine	Liaoning	Shenyang	Ping Hou
Beijing Tsinghua Changgung Hospital	Beijing	Beijing	Ping Zhang
Guizhou Provincial People's Hospital	Guizhou	Guiyang	Qiang Wu
The First Affiliated Hospital of Xiamen University	Fujian	Xiamen	Qiang Xie
Quanzhou First Hospital	Fujian	Quanzhou	Rong Lin
Wuzhou People's Hospital	Guangxi	Wuzhou	Shaowu Ye
The Central Hospital of Jilin	Jilin	Changchun	Shuangbin Li
Xiangya Hospital Central South University	Hunan	Changsha	Tianlun Yang
Guangzhou Red Cross Hospital	Guangdong	Guangzhou	Tongguo Wu
**Hospitals**	**Province**	**City**	**Investigator**
The First Affiliated Hospital of Guangzhou Medical	Guangdong	Guangzhou	Wei Wang

College			
The First Affiliated Hospital of Wenzhou Medical University	Zhejiang	Wenzhou	Weijian Huang
The Second Affiliated Hospital of Soochow University	Jiangsu	Suzhou	Weiting Xu
Wuhan Asia Heart Hospital	Hubei	Wuhan	Xi Su
The First Affiliated Hospital of Soochow University	Jiangsu	Suzhou	Xiangjun Yang
Affiliated Hospital of Yan'an University	Shaanxi	Yan'an	Xiaochuan Ma
The First People's Hospital of Jining	Shandong	Jining	Xiaofei Sun
The Central Hospital of Taiyuan	Shanxi	Taiyuan	Xiaoping Chen
West China Hospital of Sichuan University	Sichuan	Chengdu	Xiaoping Chen
The Third Affiliated Hospital of Guangzhou Medical College	Guangdong	Guangzhou	Ximing Chen
The First Affiliated Hospital of Wannan Medical College	Anhui	Wuhu	Xingsheng Tang
Tangdu Hospital of The Fourth Military Medical University	Shaanxi	Xi'an	Xue Li
Shanghai East Hospital Affiliated to Tongji University	Shanghai	Shanghai	Xuebo Liu
Xiamen Cardiovascular Disease Hospital	Fujian	Xiamen	Yan Wang
Zhongnan hospital of Wuhan University	Hubei	Wuhan	Yanggan Wang
Fujian Provincial Hospital	Fujian	Fuzhou	Yansong Guo
The First Affiliated hospital of Dalian Medical University	Liaoning	Dalian	Yanzong Yang
The First People's Hospital of Changde	Hunan	Changde	Yi Huang
The First Affiliated Hospital of China Medical University	Liaoning	Shenyang	Yingxian Sun
The Fourth Affiliated Hospital of China Medical University	Liaoning	Shenyang	Yuanzhe Jin
Cangzhou Central Hospital	Hebei	Cangzhou	Zesheng Xu
The Central Hospital of Shaoyang	Hunan	Shaoyang	Zewei Ouyang
The People's Hospital of Liaoning Province	Liaoning	Shenyang	Zhanquan Li
The First Affiliated Hospital of Jiamusi University	Heilongjiang	Jiamusi	Zhaofa He
Tangshan Gongren Hospital	Hebei	Tangshan	Zheng Ji
Huaibei Miners General Hospital	Anhui	Huaibei	Zhenqi Su
Linyi People's Hospital	Shandong	Linyi	Zhihong Ou
